# Workplace Health Promotion Among Ethnically Diverse Women in Midlife With a Low Socioeconomic Position

**DOI:** 10.1177/10901981211071030

**Published:** 2022-02-05

**Authors:** Marjolein Verburgh, Petra Verdonk, Yolande Appelman, Monique Brood-van Zanten, Carel Hulshof, Karen Nieuwenhuijsen

**Affiliations:** 1 University of Amsterdam, Amsterdam, The Netherlands; 2 Vrije Universiteit Amsterdam, Amsterdam, The Netherlands

**Keywords:** workplace health promotion, RE-AIM, low SEP, ethnicity, midlife, menopause

## Abstract

Workplace health promotion (WHP) may be an appropriate way to support women with a low socioeconomic position (SEP) during midlife. Little is known about reaching and engaging women in WHP, particularly not at the intersection of midlife, low SEP, and ethnicity. We initiated the ProudWoman project, in which we implemented a WHP intervention aimed at supporting midlife women as a pilot in an academic hospital. We qualitatively evaluated the implementation using the RE-AIM framework. The pilot comprised multiple steps: tailoring the intervention to the needs of ethnically diverse group of midlife women with a low SEP, developing an implementation protocol, implementing the tailored intervention, and evaluating the implementation process. The main findings of our study are: (1) due to a wide range of recruitment activities that were actively deployed, we were able to *reach* an ethnically diverse group of midlife women with a low SEP; (2) regarding *adoption*, awareness of the relevance of this topic as an occupational health challenge was not self-evident at the organizational level; (3) according to our participants, various facilitators and barriers should be taken into account in the *implementation* of the work–life program; and (4) our focus group discussion revealed as *maintenance* is relevant to these levels in different ways, awareness of midlife and menopause as an occupational health challenge should be raised at four professional levels. We conclude that elements, such as an active and personal recruitment approach, are important in the implementation of WHP for ethnically diverse midlife women with an SEP.

Workers who hold a low socioeconomic position (SEP) are more likely to have unfavorable working conditions, including a high physical and psychosocial workload, low level of autonomy, and little social support, compared with other groups of workers (Burdorf et al., 2016). This puts them at greater risk of poor health and work functioning (Burdorf et al., 2016).

In addition, in the age from 45 to 60 years old, women experience the transition to midlife, which includes both a biomedical transition and a life-phase transition.^1^ Menopause occurs at a median age of 51 years (Hardy et al., 2017). Menopause, defined as permanent cessation of menses resulting in estrogen deficiency, is associated with a wide range of symptoms that can last for many years, such as hot flashes, night sweats, sleep disturbance, loss of energy, memory loss, low mood, sexual dysfunction, and joint pain (Griffiths et al., 2013; ^Kopenhager & Guidozzi, 2015)^. Menopausal symptoms can have a negative influence on women’s work and productivity (Jack et al., 2016)—how women experience menopause transition may differ across contexts and cultures although little is known about cultural differences (Bendien et al., 2019). Previous studies have shown that factors typically associated with a low SEP are associated with the longer duration and severity of menopausal symptoms (Avis et al., 2015; Brzyski et al., 2001; Hunter et al., 1986; Polit & LaRocco, 1980). Also, menopausal transition is linked to an unfavorable cardiovascular risk profile, including the development of hypertension, diabetes mellitus, weight gain including unfavorable fat distribution, and a rise in cholesterol levels (Appelman et al., 2015). Menopausal symptoms and cardiovascular risk can be aggravated by an unhealthy lifestyle (Li et al., 2003), which is more common among people with a low SEP (Stringhini et al., 2010). Hence, the transition to midlife is not only a biomedical transition but also a life-phase transition in which women undergo social change, such as changing social roles, like becoming an informal caregiver for chronically ill or disabled parents (Hardy et al., 2017).

Workplace health promotion (WHP) may be appropriate to support women during midlife, because WHP interventions aim to improve lifestyle and consequently improving health and functioning at work (Robroek et al., 2012). WHP can be defined as the combined efforts at the level of employer, worker, and society to improve health and well-being in the workplace (Robroek et al., 2012). Despite women’s different needs and problems in the workplace compared with men, little is known about reaching and engaging women (Karnaki et al., 2008). Women have not been addressed as a separate group, and interventions do not take gender-related factors into account (Collins et al., 1997; Karnaki et al., 2008). For instance, barriers to participation in WHP experienced by women include lack of time or multiple roles and responsibilities (Campbell et al., 2002). In addition, it can be challenging to reach and engage workers with a low SEP who belong to culturally and ethnically diverse groups, because of interventions that fail to address particular needs, such as providing intervention activities in more than one language or employing the help of interpreters (Bukman et al., 2016; Glasgow et al., 1993; Hartman et al., 2013; Lakerveld et al., 2008; Teuscher et al., 2015). Tailor-made interventions could be helpful (Jansen et al., 2007), but little is known about tailor-made WHP interventions for ethnically diverse midlife women with a low SEP.

In fact, there are very few scientific evaluations of WHP interventions that address health problems of midlife women overall (Ariyoshi, 2009; Bendien et al., 2019; Hardy et al., 2018). In The Netherlands, a company called HealthyWoman has developed the work–life program (WLP). This program is aimed at supporting women during midlife in making choices that enhance health and well-being in their working and private lives. The WLP consists of three components: menopause counseling, coaching to improve work–life balance, and physical training (see Box 1). The WLP has been previously implemented in multiple Dutch work organizations. In these cases, the intervention was offered outside the workplace and working hours, and women participated in their own time. No interpreters were used to combat the exclusion of women who were insufficiently fluent in Dutch. Moreover, a culturally sensitive approach was not adopted, and both female and male WLP professionals were employed.

**Box 1.** Original Version WLP.

**Intake session** (0.5 hr) Information intervention, background, and availability of women **Intervention** Three individual components in eight sessions of 1 hr for several months, no fixed schedule, but fixed order **Menopause consultation** (1 hr) Information menopause, symptoms, treatment and referral options, diet and exercise, health check (weight, intercourse, blood pressure) **Work–life coaching** (3× 1 hr) Personal goals, information and advice on dealing with work–life balance **Physical training** (3× 1 hr) Personal strength training, understanding health benefits of physical activity **Menopause consultation** (1 hr)

*Note.* See our separate article for a more detailed description of each component (Verburgh, et. al., 2020). A quantitative analysis revealed no effect on work functioning, but did show that the program positively impacted the menopausal symptoms of participants. A qualitative analysis further revealed positive changes associated with mental empowerment in four domains: behavior, physical health, mental well-being, and in the workplace. WLP = work–life program.

To fill the gap in the literature on implementation of WHP among ethnically diverse midlife women with a low SEP, we initiated the ProudWoman project in which we implemented an accommodated version of the WLP as pilot in an academic hospital. An academic hospital fit the purpose as health care is a traditionally female sector in which a great number of women work in low-paid jobs (Payne & Doyal, 2010) and because it is a large employer of women in an ethnically diverse city. This pilot comprised several steps: tailoring the intervention to the needs of an ethnically diverse group of women with a low SEP, developing an implementation protocol, implementing the tailored intervention, and evaluating the implementation process. Our research question is a follows:

**Research Question 1:** How can we reach and engage an ethnically diverse group of midlife women with a low SEP in the implementation of this WHP intervention?

## Method

### Program Implementation

#### Tailoring the Intervention and Development of the Implementation Protocol

Prior to tailoring the intervention and the development of the implementation protocol, the first author (M.V.) held exploratory interviews with various populations—women from the intended audience (*n* = 9), organizational representatives (management, Human Resources [HR]; *n* = 8), and WLP professionals (*n* = 5)—to take into account the needs of the women in the intended audience. We recorded these interviews and only made notes on the key points of each interview which formed the basis of our implementation protocol.

See Box 1 for a brief description of the original intervention components. Two major adjustments to the physical component of the original WLP were necessary: (1) flexibility in the choice to do individual or group-based physical training, and (2) flexibility in the type of physical training—walking training or strength training.

**Figure 1.**
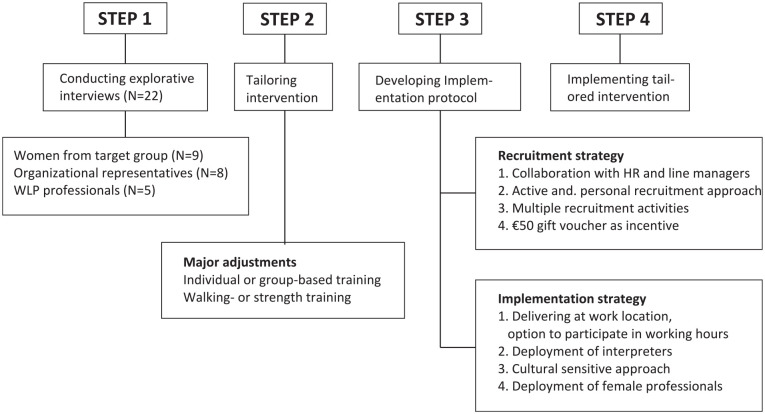
Program implementation. *Note.* WLP = work–life program; HR = Human Resources.

The implementation protocol consisted of a recruitment and implementation strategy. The recruitment strategy comprised four components: (1) collaboration with the HR department and line managers, (2) an active and personal recruitment approach, (3) multiple recruitment activities adapted to the needs of potential participants, and (4) €50 gift voucher as an incentive (to be awarded after completion of all program sessions). The planned recruitment activities comprised a personal invitation letter sent to the home address, verbal invitations issued a small group meetings in collaboration with line managers, informal information meetings, and “snowballing” during intake sessions. The implementation strategy comprised the following four components: (1) the intervention including intake session took place in the workplace with the option to participate during working hours (including intake session); (2) interpreters were deployed; (3) the approach was a culturally sensitive one in which WLP professionals were informed about the exploratory models approach that tries to understand how the social world both affects and is affected by illness (Kleinman & Benson, 2006), and we made the recruitment materials more inclusive than the original version, including photographs of women of color on the posters and flyers; and (4) female professionals were deployed (Figure 1).

#### Participants and Procedure

We recruited women aged 45 to 60 years employed in the lowest income jobs (e.g., patient food service assistants, service desk employees, and cleaners) at the Amsterdam University Medical Center, The Netherlands. The full-time annual income of this group ranges from €22,250 to €35,245. However, the majority of these women do not work at a full-time basis; thus, in reality, their income is much lower. The Amsterdam University Medical Center comprises two large academic hospitals located at two different sites. We recruited from three populations in these two hospitals. (1) We first recruited women from a number of departments at one hospital. (2) As we aimed to reach an ethnically diverse group of women, and the majority of the hospital employed women working are ethnically Dutch, we also included employees from an outsourced cleaning company. (3) Later on, the in-house services department at the second hospital was also included. The inclusion criteria were female aged between 45 and 60 years in the lowest income jobs. The following additional inclusion criteria were implemented for the women at the first hospital (excluding outsourced cleaning company employees): a contract of at least 24 hr a week with a minimum 6 months of contract remaining. With the help of interpreters, women with insufficient fluency in Dutch were also able to participate. Due to financial considerations, we did not get help from certified interpreters but recruited women from our own network who spoke Spanish, Thai, and Twi (Ghanaian language). Participation was voluntary. We outline background information on participants in Table 1.

**Table 1.** Participant Demographics (*N* = 70).

Descriptive	*n*	%	*M*	*SD*
Age			52.6	4.5
Type of contract				
Full-time	22	31.4		
Part-time	48	68.6		
Ethnicity				
Ethnic minority^a^	36	51.4		
Ethnic majority (Dutch)	34	48.6		
Educational level^b^				
Low	29	41.4		
Intermediate	36	51.4		
High	5	7.1		
SSS^c,d^			4.7	1.5
Living situation				
Alone	11	15.7		
With partner	11	15.7		
With partner and children	32	45.7		
No partner but with children	15	21.4		
Other	1	1.4		
Informal care responsibilities				
Yes	15	21.4		
No	55	78.6		
Menopausal status^e^				
Premenopause	4	7.1		
Early perimenopause	3	5.4		
Late perimenopause	3	5.4		
Postmenopause	38	67.9		
Unknown	8	14.3		

*Note.* SSS = subjective social status; CBS = Central Bureau of Statistics.

a We included 21 migration backgrounds (e.g., Moroccan, Turkish, Surinamese [Hindustani, Creole, Javanese], Ghanaian). ^b^Based on the Dutch CBS criteria, we defined low educational level by completion of primary school, lower vocational education, and lower secondary school; intermediate educational level by completion of intermediate vocational education and upper secondary school; and higher educational level by completion of upper vocational education and university. ^c^We measured SSS with the MacArthur scale, and it can be defined as an individual’s perception of his or her own position in the socioeconomic hierarchy (Tang et al., 2016). ^d^1 missing. ^e^Menopause status (*n* = 56).

Before starting the WLP, M.V. held a 1-hr face-to-face intake session with each participant individually, in which she explained the WLP in more detail, after which the WLP was customized to the needs of the woman with regard to her availability, and preferences with regard to the physical component. Subsequently, HealthyWoman contacted the woman to schedule appointments. Appointments for the group of cleaners were made using their line managers as intermediaries. A WLP professional who was a nurse specialized in menopausal support offered a menopausal consultation with each woman which was followed by work–life coaching and physical training sessions (eight sessions in total). The total lead time of the intervention for each participant was planned to last from 2 to 4 months. Recruitment of participants started in December 2018, and implementation of the program started in February 2019 and ended in January 2020. An internal implementation team responsible for tailoring, development, implementation, and evaluation was made up of researchers and clinicians from relevant disciplines, and a representative from the HR department and HealthyWoman. Although the internal implementation team was responsible for the program implementation, including tailoring the intervention and developing the implementation protocol, only the research team was responsible for evaluating the program. Although, these teams were separate, they did not operate completely independently of each other. This approach is in line with responsive evaluation (Abma, 2005) or action-based research (Cordeiro & Soares, 2018), which assumes that the research itself is an intervention in which learning from change is possible.

Throughout the program, we also collaborated with four co-creators from the intended audience to keep the internal implementation team updated about changing needs. This meant that when required practical program arrangements of the intervention could be quickly adjusted. M.V. was responsible for this collaboration and started with a pilot intake session with each of the co-creators. Moreover, M.V. planned several group meetings during implementation to explore how the group experienced the implementation process. This was part of the qualitative data that was gathered alongside implementation. Two women were asked to fulfill the co-creation role after participating in the exploratory interviews prior to implementation, and the two others were asked during recruitment after the verbal invitations issued a small group meetings in collaboration with line managers.

### Program Evaluation (RE-AIM)

#### Ethics

The research proposal was approved by the Medical Research Ethics Committee of the Amsterdam University Medical Center, location VUmc, in the Netherlands (2018.635, November 28, 2018). This study was not subject to the Dutch Medical Research Involving Human Subjects Act. Written informed consent was provided by all participants. For those insufficiently fluent in Dutch, M.V. assisted in filling out the informed consent form, or when necessary, an interpreter assisted. Individual data were treated confidentially and have not been shared with the employer. Participants were required to make arrangements with their line managers for participation during working hours.

#### Design

We used the RE-AIM framework to qualitatively evaluate the implementation of WLP at three levels—women from the intended audience, WLP professionals, and organizational stakeholders from the Amsterdam University Medical Center. Data from all three levels are included in the “Results” section. The RE-AIM framework is designed to assess health promotion interventions in “real world” settings and examine multiple dimensions: Reach, Effect, Adoption, Implementation, and Maintenance (Glasgow et al., 1999).

To establish the *reach* of the WLP, we calculated the number of women who signed up for the WLP relative to the size of the eligible target population. We also calculated the number of participants who completed the WLP. Next, in the semi-structured interviews, we assessed how participants experienced the recruitment activities. By means of a follow-up questionnaire, we assessed after which recruitment activity participants had decided to participate by simply asking them what recruitment activity had made them decided to sign up: 1 = personal invitation letter from HR to home address; 2 = information meeting; 3 = work colleagues (“snowballing”); 4 = line manager; 5 = flyer with additional verbal explanation, small gift of a fan, and tea and healthy snacks; 6 = poster; and 7 = verbal invitations issued at small group meetings (group of cleaners). We also assessed reasons for nonparticipation (mainly if these were given in response to the invitation letter and other recruitment strategies).

We examined *effect* by means of a pre- and posttest study in combination with semi-structured interviews. However, detailed information on the effect of the WLP is beyond the scope of this article and is described in a separate article (Verburgh et al., 2020).

We evaluated *adoption* by conducting a focus group discussion (FGD) with relevant stakeholders from the organization (management and HR) who were actively involved in the implementation. We inquired to what extent the organization was willing to devote attention to the themes of menopause and midlife, and adopt the WLP.

We evaluated *implementation* at the FGD with stakeholders and at the semi-structured interviews with participants and with WLP professionals. We asked about barriers and facilitators, and positive and negative experiences of the implementation.

*Maintenance* was also included in the FGD with stakeholders. This covered the extent to which the organization was willing to further take up the themes menopause and midlife, and in what way.

Additional qualitative data on all RE-AIM dimensions were collected throughout the whole implementation period of which M.V. made field notes (e.g., group meetings co-creators).

Table 2 outlines the original definitions of the RE-AIM dimensions, the definitions used in the current study, and the key data sources.

**Table 2.** Program Evaluation.

Original definitions (Glasgow et al., 1999)	Definitions in the current study	Key data sources
Reach		
Proportion of the target population that participated in the intervention	Number of participants relative to size of the target population, experiences of recruitment, reasons for nonparticipation	(1) Participation rate = number of participants who signed up for the WLP divided by the number of potential participants × 100, including number of participants who completed the WLP (2) Semi-structured interviews with women from the intended audience (3) Follow-up questionnaire (t1) (4) Additional qualitative data
Effect		
Not in the current study		
Adoption		
Proportion and representativeness of settings (such as worksites, health departments, or communities) that adopt a given program	The extent to which the organization was willing to pay attention to midlife and menopause, and adopt the WLP	(1) Focus group discussion with organizational stakeholders (2) Additional qualitative data
Implementation		
The extent to which the intervention is implemented as intended in the real world	The facilitators and barriers to the implementation	(1) Focus group discussion with organizational stakeholders (2) Semi-structured interviews with women from the intended audience (3) Semi-structured interviews with WLP professionals (4) Additional qualitative data
Maintenance		
The extent to which the intervention is sustained over time	The extent to which the organization is willing to further take up the theme midlife and menopause, and in what way	(1) Focus group discussion with organizational stakeholders (2) Additional qualitative data

*Note.* WLP = work–life program.

#### Data Collection

##### Semi-Structured Interviews

We planned semi-structured interviews with 12 participants, which is sufficient for data saturation (Guest et al., 2006). We selected participants using a purposive sampling strategy (Robinson, 2014) to include variety in age, ethnicity, educational level, type of work and contract, living situation, and menopausal status. Participants unable to speak Dutch were excluded, but participants who could speak some Dutch were included. However, we were not able to bridge the language barrier completely. Interviews were conducted mainly in Dutch by M.V. As we also wanted to gain insight into changes perceived to have been brought about by participation, we interviewed participants after they had finishing the program. The main topics were recruitment (e.g., experiences, time of decision to participate), perceived changes due to participation, and implementation (positive and negative experiences). Interviews lasted on average an hour.

In addition, M.V. held semi-structured interviews with five of the 10 professionals who had been most involved in implementation: two menopause counselors, two work–life coaches, and one physical trainer. The main topics discussed were facilitators and barriers in implementation. Long-term contact with these professionals was established throughout implementation, and interviews (average length of 30 min) were conducted by telephone.

##### FGD

After implementation was finished, we organized one FGD to reflect on implementation with six organizational stakeholders: a representative of HR involved in implementation (Stakeholder A), a facility department manager involved in starting up the ProudWoman project in her previous job (Stakeholder B); a services department manager (Stakeholder C); an HR advisor from the facility department involved in implementation (Stakeholder D), and two line managers from the cleaning company (Stakeholders E and F). We chose for one FGD with organizational stakeholders, because we only wanted to include participants who had been actively involved in this pilot. The project leader (K.N.) moderated, and M.V. took field notes. The main topics discussed were adoption of midlife and menopause and the WLP, points of concern in the implementation, and willingness to further take up the theme midlife and menopause in relation to work. The discussion lasted about 2 hr.

#### Data Analysis

We recorded and transcribed all interviews and the FGD verbatim. Two researchers (M.V. and K.N.) reviewed the data to identify the key themes in each RE-AIM dimension. Initially, after structuring the transcripts by means of the RE-AIM dimensions and related subdimensions, M.V. and K.N. inductively coded the transcripts separately—the first three interviews with participants, the first two interviews with professionals, and the FGD. They jointly compared the inductive codes for agreement, retaining only those codes for which agreement was reached; this led to a coding scheme. Subsequently, M.V. coded the other interviews according to this coding scheme, adapting it when a new code came up (Braun & Clarke, 2012). K.N. checked the new codes, after which we retained those for which agreement was reached, which led to a final version of a coding scheme structured by the RE-AIM dimensions. We used MAXQDA 2020 (VERBI Software, Berlin, Germany).

## Results

### Reach

Initially, few participants signed up after the planned recruitment activities; therefore, we expanded our recruitment activities. We promoted the ProudWoman project by distributing posters and flyers in the hospital wards. When distributing flyers, we explained about the project while offering a small gift of a fan, and tea and healthy snacks. At this point, we withdrew the additional inclusion criteria for the women at the first hospital. Therefore, we were only able to calculate the size of the eligible target population based on the original planned study population to whom the additional inclusion criteria applied. On the basis of the number of women who signed up, and the known number of the original eligible target population, we estimate that 17.3% (51 of 295) actually signed up. After expanding our recruitment activities, 19 more women signed up. In total, 70 women signed up, 51 of which completed the WLP (72.9%), four women missed one program session (5.7%), one woman missed two program sessions (1.4%), and 14 women dropped out immediately or shortly after the start (20.0%).

In Table 3, we outline the specific recruitment activity after which participants decided to participate, with corresponding quotations. Most women decided to participate immediately after the first personal invitation letter (*n* = 25). The personal invitation letter was well received as they did not frequently read email. Although most women decided to participate before the information meeting, they perceived this meeting as having additional value. The information meeting created curiosity about the WLP and provided recognition of midlife-related health issues. Some participants said that their social environment played an important role in their decision to participate, or that we never would have been able to reach them without the personal information given while distributing flyers. In particular, women with a migratory background needed further explanation of the program if they could not read or fully understand the letter. For the ethnically diverse group of cleaners, verbal invitations were organized issued at small group meetings with the support of line managers. The presence and support of line managers and other colleagues—particularly those of the same ethnic background—was important to provide a safe haven for talking about the ProudWoman project. These line managers know their workers quite well as they not only direct them in their work but often also assist with private matters. In other words, they were important key figures as they created trust.

**Table 3.** Recruitment Activities That Made Women Decide to Participate (*n* = 56).^a^

Recruitment activity	*n*	Quotation examples of experiences^b^
Personal invitation letter to home address from HR	25	“I liked the letter better because I don’t always look at my e-mail. [. . .] I liked it—the letter—because it is personal. It means you can read it again a few times.” (20191111, 56-year old Portuguese employee medical administration)
Information meeting	11	“I thought it was very positive. [. . .] Actually, it sort of felt like recognition. Because hardly anyone talks about it, and I thought oh yes, that’s right, that’s right—that’s just what it’s like! It’s great that you can finally talk about all these vague complaints you have. The ones you can’t put your finger on, not bad enough to make you stay at home but that still bother you. Some days they really slow you down. [. . .] And actually, it just being acknowledged, at a meeting like that, it was a real eye opener for me.” (20191007, 58-year-old native-Dutch employee, Central Sterilization Department)
Work colleagues (“snowballing”)	5	“But I thought, well I won’t open it straight away, but I had it [envelope containing personal invitation letter]. I thought, I’ll open it sometime. I don’t always open post that isn’t important straight away, you know? [. . .] So I came to work and on the corridor I heard, I saw some colleagues who were talking about it. And they were saying that it was a good thing and maybe we’d learn something too. And then I thought, you know what, I’m going to do it too.” (20101127, 48-year-old Surinamese patient food services assistant)
Line manager	3	“I got it at my home address and I also got an e-mail from my line manager. [. . .] Yes, she was pleased too. She said she thought of me straight away, because she knows about my situation. She also felt a bit sorry for me, because of how my health wasn’t too good. So when she saw this, she thought: Yes, it is just right for you. So then, after that I had my say. And then she said: have you read your e-mail? I said yes, and since then I have had a letter at home. And I said: I signed up straight away. And then she said: yes, that’s really great.” (20200123, 54-year-old Surinamese patient food services assistant)
Flyer with verbal explanation, small gift of a fan, and tea and healthy snacks	2	“[. . .] a paper [the personal invitation letter], I find it more difficult. [. . .]. And then it was only the second time [after flyer and verbal explanation], and then the penny dropped, oh I get it! Now I understand.” (20191104, 54-year-old Brazilian patient food services assistant)
Poster	2	No quotation available
Verbal invitations issued a small group meetings (group of cleaners)	8	No quotation available

*a*Information is gained from follow-up questionnaire (t1) which was filled out by 56 participants. HR = Human Resources.

b Quotations provided are from the participants from the 12 semi-structured interviews.

Reasons for nonparticipation were lack of time, inability to leave the work floor, availability of WLP appointments did not match their own planning, rooms where the program sessions were held were too far away from their own ward, too few menopausal symptoms to follow this fairly intensive program, and not knowing what the program entailed.

### Adoption

Despite the willingness of organizational stakeholders to pay attention to midlife and menopause, and to adopt the WLP, it emerged from the FGD that the stakeholders were unaware of the impact that menopausal transition can have on work. During the implementation of the program itself, it soon became clear to the organizational stakeholders why attention to this topic was relevant: Women suffer from menopausal symptoms, 25% of their employees are women aged 45 years or older, sickness absence is much higher in this group than among other employees, and the subject is still taboo in the workplace. At times, the organizational stakeholders received negative responses from colleagues at the organizational level. They needed to overcome some barriers to convince colleagues that menopause is relevant to work and requires WHP.

### Implementation

We identified facilitators and barriers at the level of women in the intended audience, WLP professionals, and organizational stakeholders. In Table 4, the facilitators and barriers with corresponding quotations at each level are presented.

**Table 4.** Facilitators and Barriers According to Women From the Intended Audience, Professionals, and Organizational Stakeholders.

Facilitators	According to . . .	Quotation examples
1. WLP at work location, with option to participate during working hours	• Women	“I always do it if I have day shift, or if I have late shift then I come to do the session first, and after that I get on with my work, or after the session, an hour’s break, then I go straight back to work. Then it is a like a part of your day. But imagine if I had to come all the way here from home every day, or from another city then I’d think—just forget it. No, I wouldn’t do it then . . .” (20100924, 54-year-old Brazilian patient food services assistant)
	• Professionals	“Yes, this is a completely different target group. I don’t get women like these in my own practice. They wouldn’t know of my existence.” (20200206, menopause counselor)
	• Organizational stakeholders	“[. . .] I still believe that this should be on offer on the work floor because I hope that in time it will work preventatively and not result in absence due to sickness. And that these people . . . well, that they will feel better in themselves anyway. Even that on its own would be an improvement.” (20200204, Stakeholder A)
2. Tailor-made intervention	• Professionals (physical trainer)	“Now, I found the large groups rather difficult. Because it is difficult to pass on as much information as you would want to [. . .] Often it was more of a social event for the ladies, but then again, they did join in enthusiastically and even though we couldn’t do as much as you can in a one-on-one session, I get the idea that ultimately they picked up some information and were able to get something out of it for themselves. But from my viewpoint, the large groups were the most challenging . . . yes, indeed. In that situation you can’t give the individual attention that you would like to.” (20200114, physical trainer)
	• Women	“I thought the work-life coach was really important, because in one-on-one, then we talk about the question of what did I think—group discussions or one-on-one? I like individual, because you can really say what you mean. You can say anything you want, and she really pays attention to you.” (20100924, 54-year-old Guinean patient food services assistant)
	• Organizational stakeholders	“[. . .] It could indeed be a bit more tailor-made; that would be good because there are elements that may be important to someone, but there again may not be. And you have to go right through it.” [. . .] “And then you may run a slight risk that they may avoid things that are actually very important and should really be discussed.” (20200204, Stakeholder C)
3. Forms of practical support	• Professionals	“I think that the level of education is not that important in coaching. It’s a fact that the higher the level of education that people have, the more often they want an explanation supported by theoretical models, so everyone learns in a different way. [. . .] Explaining by means of theoretical models isn’t a lot of use to people who haven’t had much education. But a good way is to use pictures, or to sketch models and then—then you actually achieve the same results that you do with people with more education.” (20200114, work–life coach) “I remember two different interpreters. [. . .]. Neither of them knew any of the women I was counseling, but they became very involved. And they found the subject interesting, certainly one of the interpreters found the subject really interesting and said that she had also learned something from it. That was quite funny. [. . .]. They were both women, I think this is absolutely necessary. It was quite striking that they didn’t only talk to me, but they really made contact with the woman.” (20200206, menopause counselor)
4. Female professionals	• Women	“With men? No. [. . .] There are so many things you just can’t tell a man, but with women, yes, you can tell them a lot of things and . . . not be ashamed either.” (2019119, 47-year-old Moroccan department assistant)
Barriers	According to . . .	Quotation examples
1. Option to participate during working hours	• Women	“No, because I went to a reasonable number of sessions outside my working hours and then she [line manager] even made a comment about it, something like: ‘But, you didn’t have to do that,’ you know what I mean? But I thought to myself, yes, well you know, I know how difficult it is sometimes to fill all the shifts on our roster.” (20191218, 47-year-old native-Dutch service employee)
	• Professionals	“It sometimes caused a bit of a problem, because the women felt that it meant they were letting their colleagues down, or if the department was really busy they thought that leaving the department wasn’t really the right thing to do. On the other hand, some women thought it was great. That when it got really busy, they were able to take a break to catch their breath. So generally, yes, very pleased that it was possible.” (20200114, physical trainer)
	• Organizational stakeholders	“Yes, it was quite a problem for us. We have some ladies who have more than one job, and . . . caring to do at home. We allowed them to go in working hours, and . . . and sometimes it went wrong. They thought that they weren’t allowed [to go], or they didn’t want to go.” (2020024, Stakeholder F)
2. Failure to follow clear guidelines for duration between program sessions	• Professionals	“It was important to have a certain rhythm in the sessions, so if you going to conduct three sessions with someone then it should be at a frequency of one every three weeks. Rhythm and regularity helps in this. It also creates calm which creates clarity which promotes more progress, but sometimes there was much too much time between sessions.” (20200114, work–life coach)
	• Women	“I thought it was a shame that there was such a long time between sessions, but . . . but what it isn’t there, isn’t there. [. . .] Yes, it’s a shame, I mean the walking training, I had that in May and I thought there was too much time between, you know, then and the second time.” (20191007, 58-year-old native-Dutch employee Central Sterilization Department)
3. Problems with availability and findability of rooms	• Professionals	“A few times we had to pick up and move during a session. Or the location was unavailable. [. . .] It was quite stressful because it meant that they couldn’t find me.” (20200214, work–life coach)
	• Women	“It would be better for us if we only had one place, not only me, lots of people were searching, searching, searching.” (20191104, 54-year-old Brazilian patient food services assistant)

*Note.* WLP = work–life program.

#### Facilitators

The first facilitator was that the WLP took place in the workplace, with the option to participate during working hours. This made participation accessible and feasible. It provided the opportunity to save time and money (e.g., travel expenses). Various participants said that they would not have participated outside working hours, mainly because they have caring responsibilities in their private lives, so they preferred not to participate outside working hours. The second facilitator was that the WLP was tailor-made mainly with regard to the physical component. The flexible format in terms of type of physical training and having the option to take part in individual and group-based training was perceived positively, in particular by women with a non-Western migratory background. Not all women had experience of physical exercising. Strength training appeared rather ambitious, and walking training was better suited to their needs. In addition, a number of women strongly preferred to train in a group setting. Some Muslim women did not want their families to know about their participation in the WLP. By training in a group with colleagues, they could avoid being seen alone with an individual trainer. However, knowledge transfer in a group setting was experienced as challenging by the physical trainer. Almost all participants preferred an individualized approach to the other two program components, as they could express themselves and get personal attention. And although the physical component was flexible in its content, the organizational stakeholders stated that the number of sessions per WLP component should be more flexible to accommodate individual needs. Simultaneously, this was also seen as a risk by the stakeholders, in that women avoid important issues that they experience as difficult, such as improving lifestyle by doing physical exercises. The third facilitator was that varying forms of practical support were provided for low literacy level and language barrier. Not only the videos and illustrations were appreciated but the support of interpreters was well received by professionals as they were very involved, the richness of the sessions was not lost, and the facts that they were all women and physically present gave added value. The fourth facilitator was that only female professionals were deployed. This was particularly relevant to the work–life coaching sessions, where women with a non-Western migratory background would rather not discuss sensitive topics with male professionals.

#### Barriers

The first barrier was having the option to participate during working hours as this sometimes proved to be difficult in practice. This often occurred when the wards were busy and finding a replacement was challenging, or when participants felt a great sense of responsibility toward colleagues or patients. The second barrier was the failure to follow clear guidelines for duration between program sessions which sometimes resulted in a longer or shorter intervention lead time than planned. Some professionals experienced this change in lead time as a barrier to making progress, because it hindered putting new insights gained from the program sessions into practice, and the timing of evaluation. The third barrier was problems with the logistics, in particular the availability of meeting rooms. At one hospital, appointments were spread over different departments which resulted in participants regularly arriving at their appointment either in a hurry or late. In addition, various work–life coaching sessions were interrupted, because the rooms had been double-booked. It also had a negative effect if rooms were too far away from the workplace.

### Maintenance

All organizational stakeholders agreed on the importance of raising awareness of midlife and menopause as an occupational health challenge at the Amsterdam University Medical Center. This should be done at four professional levels—employees, line managers, HR advisors, and occupational health physicians—because this subject is relevant to these levels in different ways. For example, implementing a work-related intervention, particularly one offered during working hours, requires that before she will sign up for an intervention, an employee recognizes that the challenges she experiences are related to menopause and midlife. Next, line managers must acknowledge this occupational health challenge to facilitate the participation of their employees in program sessions during working hours. Finally, when menopause-related sick leave or other midlife-related challenges occur, HR advisors and occupational health physicians need to be aware and knowledgeable about suitable support and treatment options. The organizational stakeholders advised organizing low-threshold meetings which means public group meetings (at lunchtime, for instance) where information is given in an accessible way, as well as teaching line managers how to discuss the topic in a helpful way, facilitating permanent low-threshold support—such as bringing a menopause counselor into the organization to make this support more easily accessible. The latter suggestion is particularly important in reaching ethnically diverse women for whom the step to see their general practitioner may be quite big. One obstacle to permanent low-threshold support is its high cost that departments may not be able to pay. Furthermore, in raising awareness, it is important to not problematize midlife and menopause, but normalize it by using role models and a fun factor. The organization has now permanently included this topic in its HR policy; there is a menopause consultant, and annual low-threshold meetings are organized.

## Discussion

The main findings of our study are as follows: (1) due to a wide range of recruitment activities that were actively deployed, we were able to *reach* an ethnically diverse group of women in midlife with a low SEP; (2) regarding *adoption*, awareness of the relevance of this topic as an occupational health challenge was not self-evident at the organizational level; (3) according to our participants. various facilitators and barriers should be taken into account in the *implementation* of the WLP. These include intervention taking place in the workplace with the option to participate during working hours, tailor-made interventions, varying forms of practical support, and female professionals; (4) our FGD revealed as *maintenance* is relevant to these levels in different ways, awareness of midlife and menopause as an occupational health challenge should be raised at four professional levels: employees, line managers, HR advisors, and occupational health physicians.

Our study has yielded a great deal, in particular with regard to reach. With our 17.3% participation rate, we have by no means reached the whole target population. However, comparing this number with that of a previous report on WHP participation rates stating that rates vary from 8% to 97% (Bull et al., 2003), we did not do too badly. We were able to reach this number by using an active recruitment strategy, including personal contact without using the same recruitment activity for every woman. Trust was key in recruiting the group of cleaners for whom collaboration with line managers was required. This key factor is supported by another study reporting that trust is important in the recruitment of people of a low SEP and of different ethnic origins (Teuscher et al., 2018). Another important key factor was making real contact, as in coming together in a group setting in which women were given space to share their own personal experiences to start a process of recognition. Therefore, recruiters should not remain detached but need to take an active approach in which they establish personal relationships with the relevant stakeholders and the target population. In particular, in the recruitment of women from ethnic minorities, a traditional approach with a distanced recruiter is not to be recommended (Levkoff & Sanchez, 2003), and proximity should be pursued.

This process evaluation made clear that an intervention should be tailor-made, in particular when it comes to taboo subjects. As previously advocated (Peersman et al., 1998), there is no such thing as an one-size-fits-all intervention. For instance, if we had not embedded a group-based option in the physical component, we would have lost some of the Muslim women. Furthermore, a tailor-made intervention does not mean ruling out protocols. In fact, protocols may enhance tailoring as they are localized and contextualized (Jansen et al., 2007). We advocate an implementation protocol in which flexibility is embedded to address different needs.

Offering the WLP in the workplace has been important in reaching and engaging these women. However, when scheduling program sessions during working hours, we identified various barriers that could be explained by various reasons. First, these women work in Dutch health care and in underresourced professions doing shift work with tight working schedules. For quite some time, there has been a chronic shortage of staff (University of West Virginia, 2020). Second, women often have many other informal caring responsibilities. This makes women less flexible about working longer to finish their work because of leaving their ward for a program session. Third, the importance of this topic was not sufficiently acknowledged by the different organizational levels, and therefore practicalities around the intervention were insufficiently facilitated.

Employers can play an important role in overcoming these barriers. To involve line managers in implementing the program, awareness of the relevance of this topic must first be raised. This is necessary to promote an openly supportive attitude toward the intervention. When raising awareness, it is important to normalize this subject. This is in line with a study that explored women’s perspectives on employers and line manager support (Hardy et al., 2017). Second, the program must be very well tailored to the work schedules of these women, as they generally do not have access to an office in which to meet. This requires employers to provide the right practical facilities, such as enough rooms in which program sessions can be held. In the implementation of the WLP at the first location, the scarcity of rooms in the Amsterdam University Medical Center left only few places where groups could be organized.

### Limitations and Strengths

We did not adopt a traditional approach in which M.V. was detached from the study population. This proximity was a strength, as it was used as a strategy to reach and engage women (e.g., personal contact in recruitment, informal conversions throughout the program). However, proximity is not necessarily “in line” with traditional epistemologies stating that researchers should be detached from the intervention itself. Therefore, proximity can also be perceived as a limitation of this study. In this study, the role of M.V. is comparable with the so-called Hawthorne effect which is defined as increased impact produced by the psychological stimulus of being singled out and made to feel important (Bloombaum, 1983). Furthermore, proximity may have caused social desirability bias (Collins et al., 2005) which can also be seen as a limitation, meaning that participants were potentially more positive about the WLP than if the researcher had been more detached. What we did to prevent social desirability bias was to emphasize at the beginning of the interviews that the research team was a separate party from the intervention provider, and did not have any interest in the research outcomes. As we did not include women unable to speak Dutch in the interviews, another limitation of this study is that we did not completely bridge the language barrier. Our study may have missed important insights from potential participants who would have needed of an interpreter. However, we also wish to stress that the language barrier can be bridged not only by language. Communication takes place in different ways and forms. Thus, despite the language barrier, we suspect that communication between professional and participant went well through mutual effort. Consequently, we think that the missed insights are limited.

### Implications

This process evaluation has helped to gain insight into how an ethnically diverse group of women with a low SEP can be reached and engaged in a WHP intervention aimed at supporting women during midlife. We conclude that six elements are important for the implementation of WHP among these women. First, the recruitment strategy must take an active and personal approach. Second, the intervention should take place at the workplace with the option for participation during working hours. Third, the intervention should be tailor-made. Fourth, varying forms of practical support should be provided. Fifth, female professionals should be deployed. Sixth, awareness of midlife as an occupational health challenge should be raised at different levels in the organization.

We recommend to adapt the implementation of WHP interventions for ethnically diverse midlife women with a low SEP taking these six elements into account. Implementing among an ethnically diverse group of women with a low SEP is a challenging task but certainly not impossible. It requires a different strategy than for other groups of workers.

## Notes

1. We acknowledge that there is a debate about definitions and measures as regards sex and gender. Not all women menstruate or go through menopause, and not all people who menstruate are women. In this study, we use “women” to refer to those people who identify as women and who go through menopause.
